# Analysis of the Incidence and Severity of Cellulitis During the COVID‐19 Pandemic in Japan

**DOI:** 10.1111/1346-8138.17853

**Published:** 2025-07-14

**Authors:** Tomoyo Sato, Kazuhiro Abe, Atsushi Miyawaki, Hirofumi Ohnishi, Hisashi Uhara

**Affiliations:** ^1^ Department of Dermatology Sapporo Medical University School of Medicine Sapporo Japan; ^2^ Division of Public Health Department of Social Medicine, Sapporo Medical University School of Medicine Sapporo Japan; ^3^ Department of Public Health Graduate School of Medicine, The University of Tokyo Tokyo Japan; ^4^ Department of Health Care Policy Faculty of Medicine, Hokkaido University Sapporo Japan; ^5^ Department of Health Services Research Graduate School of Medicine, The University of Tokyo Tokyo Japan; ^6^ Department of Clinical Epidemiology and Health Economics School of Public Health, The University of Tokyo Tokyo Japan

**Keywords:** cellulitis, COVID‐19 pandemic, hospital administrative data

## Abstract

During the COVID‐19 pandemic, a decline in various infectious disease cases was observed. However, changes in dermatological infectious diseases, particularly cellulitis, and the potential impact of delayed consultations on severe cases have not been fully explored. To investigate changes in the number of cellulitis patients and severe cases during the COVID‐19 pandemic. We employed a difference‐in‐differences (DID) design using a de‐identified claims database from 242 acute‐care hospitals across Japan to compare the pre‐pandemic period (January 1, 2015, to December 31, 2019) with the pandemic period (January 1, 2020, to December 31, 2020). The national state of emergency, declared by the Japanese government in April 2020 in response to COVID‐19, was treated as an exogenous shock. The study analyzed outpatient, inpatient, and total cases, sepsis and bacteremia complications, ambulance transport rates, length of hospital stay, and inpatient comorbidities. A total of 28 673 cellulitis cases were analyzed (24 256 from 2015 to 2019; 4417 in 2020). Severity indicators included hospitalization (8.2%), sepsis (4.1%), bacteremia (1.7%), and ambulance transport (17.0%). In the DID analysis, a significant decrease was observed in total cellulitis cases (incidence rate ratio [IRR]: 0.91; 95% confidence interval [CI]: 0.85–0.97), outpatient cases (IRR: 0.92; 95% CI: 0.86–0.98), and inpatient cases (IRR: 0.81; 95% CI: 0.66–0.99). No significant differences were found in sepsis (IRR: 0.53; 95% CI: 0.26–1.10), bacteremia (IRR: 0.73; 95% CI: 0.19–2.86), ambulance transport (IRR: 0.81; 95% CI: 0.50–1.29), or length of hospital stay (IRR: 0.83; 95% CI: 0.66–1.03). During the pandemic, the number of cellulitis cases treated in Japanese acute‐care hospitals decreased without a significant rise in severe cases, suggesting the possibility that avoidance of medical consultations may not have worsened outcomes. Pandemic‐related behavioral changes may have contributed to the reduced incidence.

## 1 Introduction

The COVID‐19 pandemic, caused by the SARS‐CoV‐2 virus, has had profound effects on global public health, not only directly through the virus itself but also indirectly by changing the patterns of other infectious diseases [1, 2, 3, 4, 5]. A notable observation during this pandemic has been the suppression of several respiratory and non‐respiratory infectious diseases. In the United States in 2020, respiratory infections such as influenza, human metapneumovirus, respiratory adenovirus, rhinovirus, and enterovirus showed a significant decline compared with pre‐pandemic levels [2].

In addition to respiratory diseases, the incidence of several non‐respiratory infections also decreased during the pandemic. Reports indicated declines in non‐allergic conjunctivitis, hepatitis C, legionellosis, and gastroenteritis caused by norovirus and rotavirus [3, 4]. In Japan, a study showed that infections such as influenza, respiratory syncytial virus, pharyngoconjunctival fever, group A streptococcal pharyngitis, chickenpox, hand, foot, and mouth disease, erythema infectiosum, herpangina, epidemic keratoconjunctivitis, mumps, *Mycoplasma pneumoniae* pneumonia, infectious gastroenteritis, hepatitis A, pertussis, rubella, and measles were significantly suppressed during the pandemic compared with the pre‐pandemic period [5]. The stringent prevention measures implemented to curb the spread of COVID‐19, including the widespread use of masks, handwashing, the use of hand sanitizers, and social distancing measures, have been credited with these reductions [5]. However, there is limited information on how the pandemic has influenced dermatological infectious diseases, particularly cellulitis.

Cellulitis is a common bacterial skin infection that affected approximately 21.2 million people worldwide in 2015 [6] and resulted in approximately 16 900 deaths worldwide [7]. Clinically, cellulitis presents with poorly demarcated erythema, swelling, warmth, and tenderness of the affected area. The most common causative organisms are group A *Streptococcus* and *Staphylococcus aureus* [8]. While cellulitis typically responds well to appropriate antibiotic therapy and is rarely fatal [9], delayed diagnosis or inadequate treatment can result in severe complications, including necrotizing fasciitis, abscess formation, septic shock, and death in extreme cases [10, 11, 12]. Timely diagnosis and treatment are therefore essential to prevent such serious outcomes.

The behavioral changes brought about by the COVID‐19 pandemic [13] may have influenced the epidemiology of cellulitis in multiple ways. The reduced frequency of outings due to stay‐at‐home measures [13] could plausibly have resulted in a decreased incidence of trauma‐induced cellulitis. Conversely, concerns over potential COVID‐19 exposure in healthcare settings [14, 15] may have led to delays in seeking medical care, thereby increasing the likelihood of presenting with more severe cases of cellulitis. Despite its clinical significance, the limited evidence supporting these hypotheses suggests a need for further research into the pandemic's impact on the incidence and management of cellulitis.

To address these gaps, this study aimed to examine the effect of the COVID‐19 pandemic on the number of cellulitis cases in both outpatient and inpatient settings by applying a difference‐in‐differences (DID) design to a nationwide hospital claims database in Japan. We also evaluated complications and comorbidities associated with hospitalization, the proportion of cases requiring ambulance transport, and the length of hospital stay to examine the change in disease severity.

## 2 Methods

### 2.1 Study Design, Participants, and Data

This study utilized a DID design to analyze the effect of the pandemic on healthcare utilization for cellulitis. The DID approach compares changes in outcomes before and after a policy intervention—in this case, the declaration of a national state of emergency by the Japanese government in April 2020—between a treatment group (the pandemic period) and a control group (the pre‐pandemic period). The pre‐pandemic period was defined as the years 2015 to 2019, while the pandemic period included the year 2020. The declaration of a national state of emergency, announced by the Japanese government in April 2020, was considered an exogenous shock [16], as it was accompanied by strong public health recommendations to stay home, practice social distancing, wear masks, and maintain hand hygiene to prevent the spread of COVID‐19 [17].

The study utilized de‐identified hospital claims data from 242 acute‐care hospitals across Japan, compiled by Medical Data Vision Co. Ltd. (Tokyo, Japan) [18, 19]. This dataset, which represents approximately 11% of all hospital admissions in Japan, includes a wide range of demographic and clinical variables and has been shown to have distributions of patient age, sex, and primary diagnosis that are representative of national estimates based on the Patient Survey conducted by the Ministry of Health, Labour, and Welfare [19]. For this study, we used a random 5% sample of patient data from the hospitals included in the dataset, spanning from 2015 to 2020. The data were aggregated on a monthly basis, and no missing data were reported.

### 2.2 Primary Outcomes

The primary outcomes of interest in this study were the total number of cellulitis cases, including outpatient visits and unscheduled hospital admissions. Cellulitis cases were identified using the *International Statistical Classification of Diseases and Related Health Problems, Tenth Revision* (ICD‐10) codes: L03.0–L03.9, L04.0–L04.9, L08.0, L08.8–L08.9, L88.0–L88.9, and L98.0 [20].

In outpatient settings, cellulitis diagnoses were limited to confirmed cases, with suspected cases excluded from the analysis. To identify newly diagnosed cases of cellulitis, we utilized the starting date of the diagnosis as recorded in the hospital database. Although the starting date may include cases in which the patient was transferred from another medical facility, it is unlikely that cellulitis would cause a patient to be transferred from one outpatient clinic to another, as cellulitis is a relatively common condition that can typically be managed in most acute‐care hospital outpatient settings.

Hospitalization data included diagnostic codes for the admission‐precipitating diagnosis, primary diagnoses, conditions requiring the most medical resources, and any complications and comorbidities present at the time of admission. Cellulitis cases were identified based on the admission‐precipitating diagnosis [21], and there were no missing values for this variable.

### 2.3 Secondary Outcomes

Secondary outcomes included the proportion of cellulitis cases complicated by sepsis (ICD‐10 codes: A40.0–A41.9) or bacteremia (A49.9), the proportion of cases requiring ambulance transport, the length of hospital stay, and the proportion of comorbidities among hospitalized cellulitis patients. The proportion of sepsis and bacteremia cases, as well as the proportion of cases requiring ambulance transport, was calculated by dividing the number of applicable cases by the total number of hospitalizations per month.

Furthermore, to investigate the actual conditions of cellulitis patients during the pandemic in greater detail, comorbidities associated with cellulitis‐related hospitalizations were examined. They included local comorbidities such as edema (I89.0–I89.9, I97.2, I60.0–I60.9), trauma (S00.0–T14.9), ringworm (B35.0–B35.9), venous disease (I87.2, I80.0–I80.9, I83.0–I83.9), decubitus (L89.0–L89.9), and dermatitis (L20.0–L30.9). Systemic comorbidities included diabetes (E10.0–E14.9), kidney disease (e.g., glomerular disease, renal tubulointerstitial disease, kidney failure) (N00.0–N19.9), liver disease (e.g., liver failure, chronic hepatitis) (K70.0–K77.9), heart failure (I50.0–I50.9), and cancer (C00.0–C97.9).

### 2.4 Adjustment Variables

For hospitalized patients, adjustment variables were patient characteristics, including age at admission, sex, and the Elixhauser comorbidity index, which adjusts for the presence of multiple comorbid conditions [22, 23]. For outpatient and total cases, age and sex at the time of diagnosis were used as adjustment variables because comorbidity data were not available.

### 2.5 Statistical Analysis

First, we described monthly statistics of cellulitis cases in pre‐pandemic and pandemic periods. Second, a multivariable Poisson regression model with robust estimators was used to assess the statistical significance of the DID analysis. The model included interaction terms between the pandemic and pre‐pandemic periods, as well as between the periods before and after April, to evaluate whether the trends in cellulitis cases changed after the declaration of a national state of emergency. We adjusted for covariates. Third, to examine dynamic changes in the incidence of cellulitis over time, monthly incidence rate ratios (IRRs) with 95% confidence intervals (CIs) were calculated using modified Poisson regression (Method S1). March (the final pre‐emergency month) was used as the reference month for comparison, and monthly IRRs were plotted.

All statistical analyses were performed using Stata software version 16 MP (StataCorp LLC), and statistical significance was set at a two‐sided *p* < 0.05.

### 2.6 Ethical Considerations

This study utilized de‐identified data from an existing hospital database, and no personal identifying information was collected. Given the anonymized nature of the data, the need for informed consent was waived. The study protocol was approved by the Ethics Review Committee of the University of Tokyo (No. 2021080NI) and the Ethics Committee of Sapporo Medical University School of Medicine (No. 6‐1‐79).

## 3 Results

### 3.1 Monthly Statistics of Cellulitis Cases

A total of 28 673 cellulitis cases were included in the analysis, of which 24 256 occurred between 2015 and 2019, and 4417 occurred in 2020. Among these, 26 326 were outpatient visits, and 2347 were hospital admissions. Throughout the entire period, 97 (4.1%) were complicated by sepsis, 40 (1.7%) by bacteremia, and 399 (17.0%) required ambulance transport among the hospitalized cases. The average length of hospital stay was 15.3 days.

Table 1 shows that the total number of cellulitis cases, both outpatient and inpatient, was higher between April and December than between January and March in both the pre‐pandemic and pandemic periods. Although the proportions of ambulance transport and sepsis complications, as well as the average length of hospital stay, were higher between April and December in the pre‐pandemic period, they decreased during the pandemic. The covariates used in the analysis are detailed in Tables S1–S3.

**TABLE 1:** Monthly statistics of cellulitis cases during the pre‐pandemic and pandemic periods.

	Pre‐pandemic (2015–2019), mean (SD)		Pandemic (2020), mean (SD)	
	January to March	April to December	January to March	April to December
No. of total patients	352.2 (23.7)	421.6 (57.4)	346.0 (19.5)	375.4 (65.9)
No. of outpatients	326.3 (22.3)	385.6 (50.3)	319.3 (20.8)	346.9 (57.6)
No. of inpatients	25.9 (6.1)	36.0 (9.2)	26.7 (1.5)	28.6 (9.5)
Complications^a^				
Sepsis, %	3.5 (3.1)	4.3 (3.1)	5.0 (2.1)	4.0 (4.4)
Bacteremia^a^, %	1.1 (2.1)	1.4 (1.9)	2.7 (4.6)	4.0 (3.1)
Local comorbidities^a^				
Edema, %	0.9 (1.6)	2.6 (2.5)	1.3 (2.3)	2.0 (2.5)
Trauma, %	5.1 (3.3)	4.8 (3.3)	9.9 (7.6)	5.6 (4.6)
Ringworm, %	3.5 (3.5)	5.0 (3.9)	2.5 (2.2)	5.9 (6.1)
Venous disease, %	4.5 (4.7)	3.5 (3.4)	6.2 (1.9)	7.0 (4.4)
Decubitus, %	1.8 (2.9)	2.3 (2.0)	3.7 (6.4)	2.7 (2.3)
Dermatitis, %	7.0 (5.3)	7.6 (4.6)	3.8 (0.2)	7.0 (5.0)
Systemic comorbidities^a^				
Diabetes, %	20.2 (8.0)	23.1 (7.7)	16.3 (2.1)	23.2 (5.6)
Kidney disease, %	5.8 (4.7)	7.2 (3.6)	6.3 (2.3)	5.1 (4.3)
Liver disease, %	0.2 (0.9)	0.8 (1.5)	2.5 (4.3)	0.3 (0.9)
Heart failure, %	6.9 (3.6)	7.9 (4.0)	6.2 (5.6)	7.7 (5.2)
Cancer, %	10.0 (8.7)	7.3 (4.8)	6.3 (2.4)	9.4 (7.4)
Ambulance transport^a^, %	14.6 (8.6)	16.4 (7.1)	19.0 (10.3)	18.2 (7.8)
Length of hospital stay, days	15.2 (3.3)	15.5 (3.2)	16.7 (3.0)	14.3 (3.7)

Abbreviation: SD, standard deviation.

^a^ The proportions of sepsis, bacteremia, ambulance transport, edema, trauma, ringworm, venous disease, decubitus, diabetes, kidney disease, liver disease, heart failure, and cancer were defined as the number of applicable cases divided by the number of hospitalizations per month.

### 3.2 Difference‐In‐Differences Analysis

In a DID analysis, we compared changes in mean outcomes between the months before (January–March) and after (April–December) the emergency declaration in 2020, and the corresponding months from 2015 to 2019, prior to the pandemic. The total number of cellulitis cases (IRR: 0.91; 95% CI: 0.85–0.97), outpatient cases (IRR: 0.92; 95% CI: 0.86–0.98), and inpatient cases (IRR: 0.81; 95% CI: 0.66–0.99) all decreased (Table 2). The proportions of sepsis (IRR: 0.53; 95% CI: 0.26–1.10) and bacteremia (IRR: 0.73; 95% CI: 0.19–2.86), ambulance transport (IRR: 0.81; 95% CI: 0.50–1.29), and the average length of hospital stay (IRR: 0.83; 95% CI: 0.66–1.03) also decreased, although not significantly. Regarding comorbidities, the proportions of edema (IRR: 0.45; 95% CI: 0.09–2.18), trauma (IRR: 0.66; 95% CI: 0.29–1.52), decubitus (IRR: 0.80; 95% CI: 0.27–2.41), kidney disease (IRR: 0.64; 95% CI: 0.28–1.46), and liver disease (IRR: 0.92; 95% CI: 0.26–3.26) among hospitalized cases declined; however, none of these changes reached statistical significance (Table 3).

**TABLE 2:** Incidence‐rate ratios (95% CI) of primary outcomes estimated by the Difference‐in‐Differences approach.^a^

	Incidence‐rate ratios (95% CI)
No. of total patients	0.91 (0.85–0.97)
No. of outpatients	0.92 (0.86–0.98)
No. of hospitalizations	0.81 (0.66–0.99)

Abbreviation: CI, confidence interval.

^a^ Poisson regression analysis was performed using robust estimators for the interaction term between the dummy variable indicating pre‐pandemic and pandemic periods and the dummy variable indicating the periods before and after April, along with their respective first‐order terms and covariates.

**TABLE 3:** Incidence‐rate ratios (95% CI) of secondary outcomes estimated by the Difference‐in‐Differences approach.^a^

	Incidence‐rate ratios (95% CI)
Complications	
Proportion of sepsis	0.53 (0.26–1.10)
Proportion of bacteremia	0.73 (0.19–2.86)
Local comorbidities	
Proportion of edema	0.45 (0.09–2.18)
Proportion of trauma	0.66 (0.29–1.52)
Proportion of ringworm	1.51 (0.48–4.71)
Proportion of venous disease	1.35 (0.60–3.07)
Proportion of decubitus	0.80 (0.27–2.41)
Proportion of dermatitis	1.33 (0.69–2.57)
Systemic comorbidities	
Proportion of diabetes	1.13 (0.82–1.57)
Proportion of kidney disease	0.64 (0.28–1.46)
Proportion of liver disease	0.92 (0.26–3.26)
Proportion of heart failure	1.21 (0.45–3.25)
Proportion of cancer	1.66 (0.66–4.16)
Proportion of ambulance transport	0.81 (0.50–1.29)
Length of hospital stay	0.83 (0.66–1.03)

Abbreviation: CI, confidence interval.

^a^ Poisson regression analysis was performed using robust estimators for the interaction term between the dummy variable indicating pre‐pandemic and pandemic periods and the dummy variable indicating the periods before and after April, along with their respective first‐order terms and covariates.

### 3.3 Dynamic Changes in Incidence of Cellulitis

**FIGURE 1:**
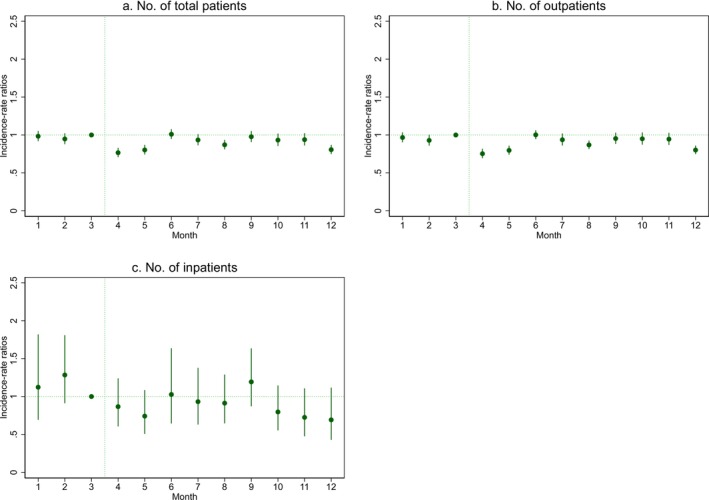
Monthly incidence‐rate ratios of cellulitis cases estimated by the difference‐in‐differences approach. Monthly incidence‐rate ratios with 95% CI were estimated by the modified Poisson regression for the interaction terms between the dummy variable for each month and the dummy variable for the pandemic period, along with their respective first‐order terms and covariates. March was set as a reference month.

Monthly changes in IRRs, as shown in Figure 1 and Table S4, revealed that the total number of cellulitis cases and outpatient visits decreased in April, May, August, and December 2020. Similarly, the number of inpatient cases declined during these months, although the changes were not statistically significant.

## 4 Discussion

Using claims data obtained from 242 acute‐care hospitals across Japan, this study found that the incidence of cellulitis decreased significantly during the COVID‐19 pandemic in both outpatient and inpatient settings. The total number of cellulitis cases decreased by 9% during the pandemic, with outpatient cases decreasing by 8% and hospitalizations by 19%, compared with the estimates assuming there had been no pandemic. The months with the most significant decreases in cellulitis cases—April, May, August, and December 2020—align with Japan's COVID‐19 waves [13, 24].

Several factors may explain the observed decrease in cellulitis cases during the COVID‐19 pandemic. First, the infection control measures implemented to prevent COVID‐19 transmission may have inadvertently disrupted the transmission of organisms responsible for cellulitis. The Japanese government's emergency declaration, which encouraged mask‐wearing, frequent handwashing, and social distancing [17], likely played a role. Cellulitis is commonly believed to occur when bacteria, such as *Staphylococcus aureus* or group A *Streptococcus*, invade the subcutaneous tissue through a disruption in the skin barrier [8]. The transmission of these pathogens may have been mitigated by the implementation of infection control measures. Furthermore, a study from Japan reported a 56% reduction in group A streptococcal pharyngitis cases during the pandemic [5], which may have indirectly contributed to the observed decrease in cellulitis cases, given that group A *Streptococcus* is a primary causative agent of cellulitis. Second, lifestyle changes induced by the pandemic, including reduced social mobility and fewer outdoor activities, may have decreased exposure to environmental risk factors for cellulitis, such as trauma and injuries. This study observed a downward trend in the proportion of cellulitis cases related to trauma, which could be explained by fewer outdoor activities during the pandemic. A study conducted in Osaka, Japan, reported a significant reduction in trauma‐related emergency evacuations during the pandemic [25]. Another study, which analyzed a trauma database of 12 general hospitals in Japan, found a 5% decrease in emergency trauma patients in 2020 compared with the 2015–2019 average [26]. Trauma, especially skin abrasions and lacerations, is a known risk factor for cellulitis [27, 28, 29], and the reduction in trauma‐related incidents may have contributed to the decline in cellulitis cases. Third, the decreases in the number of cellulitis cases may be partly attributed to patients avoiding medical consultations [14, 15]. There is a possibility that this avoidance might lead to an increase in the number of severe cellulitis cases. However, this study did not find evidence to support this concern in the context of cellulitis. The number of cellulitis cases decreased, and there were reductions in the proportion of cases with sepsis or bacteremia, the proportion of cases requiring ambulance transport, and the average length of hospital stay, though these were not statistically significant. Importantly, the decrease in cellulitis cases occurred without a significant rise in severe complications such as sepsis or bacteremia, suggesting that even though patients avoided medical consultations for cellulitis during the pandemic, it may not have worsened their condition. This also suggests that patients continued to seek timely care for cellulitis during the pandemic.

A longitudinal, single‐center study conducted at Oxford University Hospital in the UK compared the incidence of cellulitis before and during the COVID‐19 pandemic. The study included patients aged 18 years and older who presented to either the emergency department (*n* = 243 667) or acute care settings (*n* = 82 899). It was reported that hospitalizations for cellulitis decreased from 86 cases prior to the pandemic to 44 cases during the first wave of COVID‐19 [30]. However, this study was limited in scope, as it focused solely on emergency department visits and did not analyze the trends in outpatient settings, hospitalizations, or the severity of cases over time. Moreover, the study was conducted at a single center, which may limit the generalizability of its findings. Our study offers new insights into the overall number of outpatient and inpatient cellulitis cases, as well as associated complications such as sepsis and bacteremia, ambulance transport rates, length of hospital stay, and the prevalence of comorbidities in hospitalized patients. Moreover, by utilizing multicenter data and quasi‐experimental methodologies, our analysis strengthens both the internal and external validity of the findings.

Our findings have important implications for both clinical practice and health policy. From a clinical perspective, handwashing, hand sanitizing, and injury prevention may still be important for cellulitis prevention, even outside the context of an infectious disease epidemic. Healthcare providers should continue to educate their patients about these preventive measures. From a policy perspective, our results indicate that during the COVID‐19 pandemic, the system for providing care to cellulitis patients was generally maintained in Japan. These implications may help inform strategies for managing dermatological diseases in future pandemics.

### 4.1 Limitations of the Study

There are several limitations to this study. First, the DID analysis assumes that trends in the pre‐pandemic and pandemic periods are parallel. Although we controlled for known covariates, unmeasured confounding may still exist. Prior to March, however, the IRRs of the outcomes were not significantly different from 1, suggesting that the parallel trend assumption of DID was valid. Second, although the diagnostic codes used in the database are generally accurate [31], misclassification may still occur, which could affect the validity of our findings. Third, the study population was limited to patients treated in acute‐care hospitals; therefore, the findings may not be generalizable to those treated in other settings, such as clinics or non‐acute care hospitals. Patients with mild symptoms may have sought care at institutions other than acute‐care hospitals, since antibiotics are not available over the counter in Japan. However, it is worth mentioning that our results still indicated a reduction in the number of severely ill patients who had to be admitted to acute‐care hospitals. Further research using data from clinics and non‐acute care hospitals will be needed to gain a more comprehensive understanding of the cellulitis treatment trends. Finally, as this study was conducted in Japan, the findings may not be directly applicable to other countries due to differences in healthcare systems and responses to the COVID‐19 pandemic.

## 5 Conclusion

This study provides new insights into the effects of the COVID‐19 pandemic on the incidence of cellulitis in Japan. The total number of cellulitis cases decreased significantly during the pandemic in both outpatient and inpatient settings. However, trends toward reductions in the proportions of severe cases, such as sepsis and bacteremia, and the length of hospital stay were not significant. Importantly, the decrease in cellulitis cases occurred without a significant rise in severe cases, suggesting that the avoidance of medical consultations during the pandemic did not worsen outcomes. Future research should explore whether similar trends were observed in other countries and whether these reductions persisted beyond the pandemic period.

## Conflicts of Interest

The authors declare no conflicts of interest.

## Supporting information


Data S1.

## Data Availability

The data that support the findings of this study are available from Medical Data Vision Co. Ltd. (Tokyo, Japan). Restrictions apply to the availability of these data, which were used under license for this study. Data are available from https://www.mdv.co.jp/ebm/service/dataset/ with the permission of Medical Data Vision Co. Ltd. (Tokyo, Japan).
