# Supplementary material for: Analysis of the Incidence and Severity of Cellulitis During the COVID‐19 Pandemic in Japan
Source: J Dermatol. 2025 Jul 14;52(10):1512–8. doi: 10.1111/1346-8138.17853 (PMC12530467; doi:10.1111/1346-8138.17853)
Supplement: Supplementary file 1 — Data S1. [file JDE-52-1512-s001.docx]

**Supplemental materials**

**Table S1. Monthly statistics of covariates of cellulitis inpatients in pre-pandemic and pandemic periods**

|  | Pre-pandemic (2015-2019) | | Pandemic (2020) | |
| --- | --- | --- | --- | --- |
|  | **January to March** | **April to December** | **January to March** | **April to December** |
| Age, median (IQR) | 64.0 (61.0-66.5) | 68.0 (65.0-71.0) | 61.5 (56.0-77.0) | 74.0 (71.0-78.0) |
| Proportion of women, % (SD) | 49.9 (7.5) | 49.3 (6.4) | 38.8 (1.5) | 48.3 (7.8) |
| Elixhauser comorbidity index, mean (SD) | 0.90 (0.19) | 1.00 (0.12) | 0.83 (0.22) | 0.93 (0.11) |

**Table S2. Monthly statistics of covariates for cellulitis outpatients during the pre-pandemic and pandemic periods**

|  | Pre-pandemic (2015-2019) | | Pandemic (2020) | |
| --- | --- | --- | --- | --- |
|  | **January to March** | **April to December** | **January to March** | **April to December** |
| Age, median (IQR) | 58.0 (56.0-59.5) | 58.0 (56.0-59.0) | 59.5 (58.0-60.0) | 61.0 (59.0-62.0) |
| Proportion of women, % (SD) | 50.2 (2.7) | 49.5 (2.7) | 49.4 (2.7) | 49.2 (4.3) |

**Table S3. Monthly statistics of covariates for cellulitis inpatients and outpatients during the pre-pandemic and pandemic periods**

|  | Pre-pandemic (2015-2019) | | Pandemic (2020) | |
| --- | --- | --- | --- | --- |
|  | **January to March** | **April to December** | **January to March** | **April to December** |
| Age, median (IQR) | 58.0 (57.0-59.5) | 59.0 (57.0-60.0) | 59.0 (58.0-61.0) | 62.0 (60.5-63.0) |
| Proportion of women, % (SD) | 50.2 (2.7) | 49.5 (2.6) | 48.6 (2.6) | 49.1 (4.3) |

**Table S4. Monthly incidence-rate ratios (95% Confidence Intervals) of the number of cellulitis cases estimated by the difference-in-differences approach^a^**

|  | No. of total patients | No. of outpatients | No. of inpatients |
| --- | --- | --- | --- |
| January | 0.98 (0.92 - 1.05) | 0.97 (0.90 - 1.03) | 1.12 (0.69 - 1.82) |
| February | 0.95 (0.88 - 1.02) | 0.93 (0.86 - 1.00) | 1.28 (0.91 - 1.81) |
| March | Reference | Reference | Reference |
| April | 0.77 (0.71 - 0.83) | 0.75 (0.69 - 0.82) | 0.87 (0.61 - 1.24) |
| May | 0.80 (0.74 - 0.87) | 0.80 (0.74 - 0.86) | 0.74 (0.51 - 1.08) |
| June | 1.01 (0.95 - 1.08) | 1.00 (0.95 - 1.06) | 1.03 (0.64 - 1.64) |
| July | 0.93 (0.86 - 1.01) | 0.94 (0.86 - 1.02) | 0.93 (0.63 - 1.38) |
| August | 0.87 (0.81 - 0.94) | 0.87 (0.81 - 0.93) | 0.91 (0.65 - 1.29) |
| September | 0.98 (0.91 - 1.05) | 0.95 (0.88 - 1.03) | 1.19 (0.87 - 1.64) |
| October | 0.93 (0.85 - 1.02) | 0.95 (0.87 - 1.03) | 0.8 (0.55 - 1.15) |
| November | 0.94 (0.86 - 1.02) | 0.94 (0.87 - 1.03) | 0.73 (0.47 - 1.11) |
| December | 0.81 (0.75 - 0.87) | 0.80 (0.75 - 0.86) | 0.69 (0.43 - 1.12) |

1. Monthly incidence-rate ratios with 95% CI were estimated by the modified Poisson regression for the interaction terms between the dummy variable for each month and the dummy variable for the pandemic period, along with their respective first-order terms and covariates. March was set as a reference month.

**Method S1. Equations used for the estimations of the difference-in-differences approach with modified Poisson regression**

We conducted a difference-in-differences analysis using Poisson regression with robust standard errors, assuming the parallel trends and common shocks assumption.

$$\ln{(Y}_{t}) = \alpha+ \beta Treat+ \sum_{t=1}^{12} \gamma_{t} {Month}_{t}+\delta\left( Treat*{Post}_{t} \right)+ \varepsilon_{t}\ldots(1)$$

where $Y_{t}$ represents the outcome variables at month t. *Treat* indicates the treatment period (i.e., pandemic period (2020) versus pre-pandemic period (2015 to 2019)). ${Month}_{t}$ is a dummy variable for month t. ${Post}_{t}$ equals 1 if month t is April or later and 0 otherwise. α, β, γ, and δ are coefficients, with δ as our causal parameter of interest. ε represents the error term. Incidence-rate ratios were calculated as exp(δ).

Furthermore, to evaluate dynamic changes in the incidence of cellulitis cases during the pandemic, we applied the following equation using Poisson regression with robust standard errors and estimated monthly incidence-rate ratios. We plotted the incidence-rate ratios for each month calculated from δt.

$${ln(Y}_{t})= \alpha+ \beta Treat+ \sum_{t=1}^{12} \gamma_{t} {Month}_{t}+ \sum_{t=1}^{12} \delta t (Treat* {Month}_{t}) + \varepsilon_{it}\ldots(2)$$

For the total number of cellulitis patients and outpatients, and for the proportion of comorbidities, we adjusted for age and the proportion of women in equations (1) and (2). In addition, for the number of inpatients, we conducted a regression analysis, adjusting for age, the proportion of women, and the Elixhauser comorbidity index in equations (1) and (2).
